# Abnormal pupils at the bedside: rapid recognition of neurologic and systemic emergencies in acute care settings

**DOI:** 10.1186/s12245-026-01195-0

**Published:** 2026-03-25

**Authors:** Dawn C. Penney, Zoë M. Rushetsky, Donald W. Penney

**Affiliations:** 1 Cook County Hospital, 1969 W Ogden Ave, Chicago, IL 60612 USA; 2 Philadelphia College of Osteopathic Medicine, 625 Old Peachtree Rd NW, Suwanee, GA 30024 USA

**Keywords:** Pupil, Sympathetic pathway, Parasympathetic pathway, CN III palsy

## Abstract

**Background:**

Pupillary abnormalities are critical bedside findings that may indicate both benign physiologic variation and life-threatening neurologic emergencies. In emergency and urgent care settings, rapid interpretation of pupillary findings is essential for early recognition of intracranial pathology, vascular lesions, toxicologic syndromes, and ocular disease.

**Objective:**

To summarize clinically significant pupillary abnormalities encountered in acute care and propose a structured bedside framework to distinguish benign from emergent etiologies.

**Methods:**

A narrative review of the literature was conducted, focusing on pupillary neuroanatomy, examination techniques, and underlying pathophysiologic mechanisms relevant to emergency practice. Results Key conditions reviewed include physiologic anisocoria, Horner syndrome, Adie tonic pupil, Argyll Robertson pupil, relative afferent pupillary defect, Hutchinson pupil, and oculomotor nerve palsy. A practical bedside approach to anisocoria is emphasized, including identification of the abnormal pupil (dilated vs. constricted) and integration of associated neurologic findings to guide diagnostic evaluation and imaging. Emerging tools such as quantitative pupillometry and point-of-car ultrasound are highlighted as adjuncts for assessing pupillary function in complex or resource-limited settings.

**Conclusion:**

A systematic approach to pupillary examination can facilitate early detection of neurologic emergencies, guide appropriate imaging decisions, and improve clinical documentation in acute care settings.

## Introduction

The pupil is a dynamic ocular structure that serves as a critical indicator of neurologic and ophthalmologic function. Specific pupillary abnormalities may represent benign physiologic variation, while others can be the harbinger of catastrophic neurologic disease. In emergency and urgent care settings, rapid recognition and interpretation of pupillary findings can therefore provide essential diagnostic information. While many cases represent incidental findings such as physiologic anisocoria, others may signal serious pathology, including intracranial aneurysm, brainstem stroke, or impending cerebral herniation [1].

The eye has long been described as a “window to the brain,” a phrase that reflects both embryologic and physiologic reality. The retina and optic nerve originate from the diencephalon and represent direct extensions of the central nervous system [2]. Consequently, abnormalities in pupillary size and reactivity may reflect dysfunction anywhere along the visual and autonomic pathways.

The pupillary light reflex traverses several critical neuroanatomical structures including the retina, optic nerve, pretectal nuclei of the midbrain, Edinger-Westphal nucleus, and parasympathetic fibers traveling through the oculomotor nerve (CN III) to the iris sphincter muscle [2]. Because this reflex integrates both afferent and efferent pathways, abnormalities in pupillary responses can help localize neurologic injury in real time at the bedside.

This review summarizes clinically important pupillary abnormalities encountered in emergency and urgent care settings and provides a structured bedside approach for distinguishing benign findings from neurologic emergencies. Conditions discussed include anisocoria, Horner syndrome, Adie tonic pupil, Argyll Robertson pupil, relative afferent pupillary defect (RAPD), Hutchinson pupil, and CN III palsy.

## Anisocoria

### Description

**Fig. 1:**
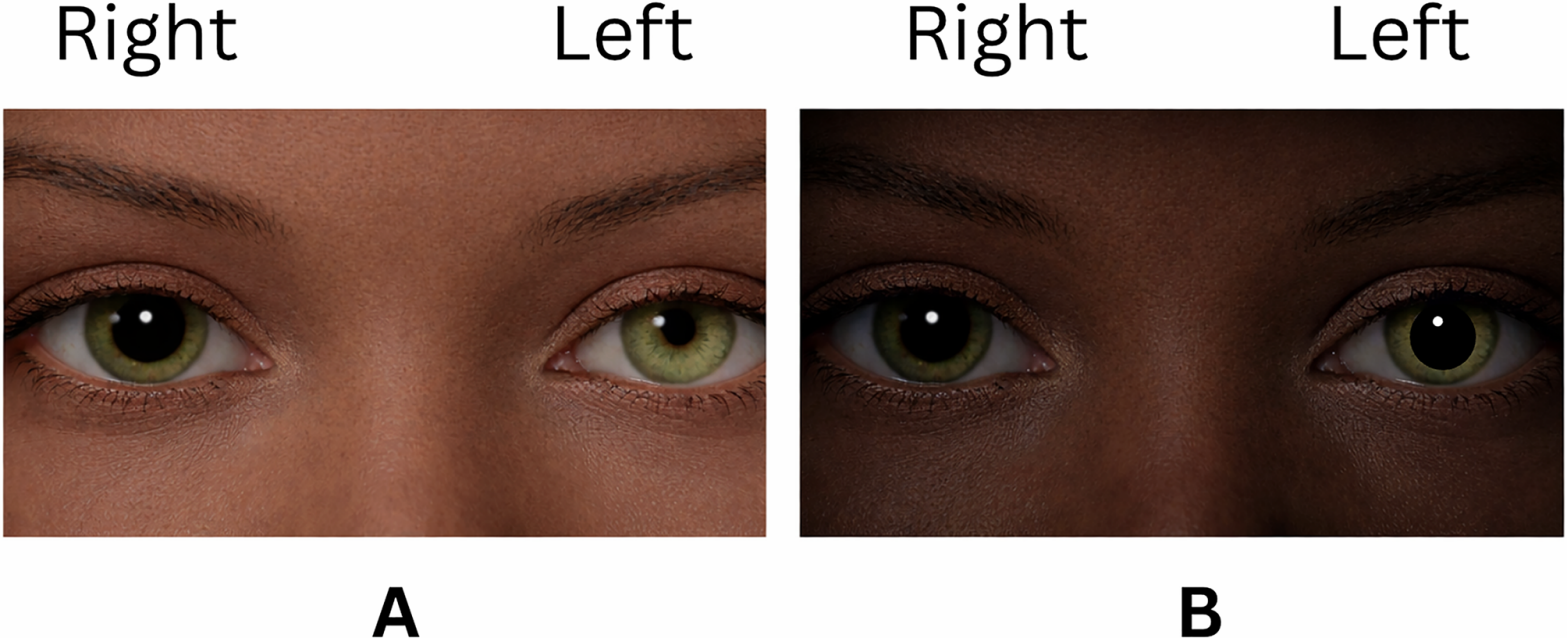
Anisocoria in ambient light and dim illumination. (**A**) In ambient light, the right pupil appears larger than the left, demonstrating anisocoria. (**B**) In dim illumination, both pupils enlarge, with the right pupil remaining relatively larger than the left. Greater anisocoria in bright conditions suggests that the larger pupil is abnormal and may indicate impaired parasympathetic-mediated pupillary constriction

Anisocoria is the most frequently encountered pupillary abnormality in emergency and urgent care settings and is defined as a difference in pupil diameter greater than 1 mm between the two eyes. Physiologic anisocoria occurs in up to 20% of the population and is typically benign; however, clinicians must determine whether the larger or smaller pupil is abnormal because this distinction guides the diagnostic approach [3]. A classic presentation of anisocoria is illustrated in Fig. 1.

### Physical examination

The key clinical question when evaluating anisocoria is which pupil is abnormal, the larger pupil or the smaller pupil, because this distinction directs the differential diagnosis and subsequent evaluation.

Evaluation of anisocoria begins by comparing pupil size in both bright and dim illumination. If anisocoria becomes more pronounced in bright light, the larger pupil is abnormal, suggesting parasympathetic dysfunction, since our pupils normally constrict in light. Conversely, anisocoria that becomes more pronounced in darkness indicates sympathetic pathway dysfunction affecting the smaller pupil, as our pupils normally dilate in the dark [3].

Emerging technologies may also assist with pupillary evaluation in challenging clinical situations. Quantitative pupillometry provides objective measurement of pupil size and reactivity and is increasingly used in neurocritical care settings to monitor neurologic status. Additionally, point-of-care ultrasound (POCUS) may allow clinicians to assess pupillary responses in patients with severe periorbital trauma or swelling when direct visualization of the pupil is not possible [3, 4].

### Neuropathophysiology

Pupil size reflects the balance between parasympathetic-mediated constriction and sympathetic-mediated dilation. Disruption of either pathway may produce anisocoria [3].

### Clinical relevance and causes

#### Dilated pupil (mydriasis)

A dilated pupil that fails to constrict normally suggests disruption of the parasympathetic pathway. Important causes include:

- CN III palsy.
- Adie tonic pupil.
- Pharmacologic mydriasis.
- Traumatic injury to the ciliary ganglion.

Pharmacologic dilation is relatively common and may occur following exposure to anticholinergic medications such as atropine, scopolamine, or inhaled ipratropium [5, 6]. Sympathomimetic agents may produce a similar appearance. Head trauma that damages the ciliary ganglion or short ciliary nerves may also result in a dilated pupil without major intracranial injury [2, 7].

#### Constricted pupil (miosis)

A constricted pupil suggests disruption of the sympathetic pathway. Important causes include:

- Horner syndrome.
- Opioid intoxication.
- Pontine lesions.
- Organophosphate poisoning.

Painful anisocoria or anisocoria accompanied by neurologic deficits should raise concern for serious intracranial pathology, including compressive aneurysm or carotid artery dissection. In these cases, prompt neurologic evaluation and appropriate imaging are required [2, 7].

### Imaging considerations

Once the abnormal pupil has been identified and the likely differential diagnosis established, diagnostic imaging should be guided by the associated clinical findings.

Horner syndrome accompanied by anhidrosis or other central neurologic findings warrants brain magnetic resonance imaging (MRI) with diffusion-weighted imaging to evaluate for brainstem stroke or other central lesions. When Horner syndrome occurs with acute neck pain or trauma, computed tomography angiography (CTA) of the neck is recommended to evaluate for carotid artery dissection. If Horner syndrome is suspected to result from an apical lung lesion, computed tomography (CT) of the chest should be obtained to assess for Pancoast tumor [8, 9].

Similarly, CN III palsy with pupillary dilation requires urgent vascular imaging, typically CTA or magnetic resonance angiography **(**MRA) of the brain, to evaluate for compressive lesions such as a posterior communicating artery (PCOM) aneurysm. When CN III palsy is accompanied by facial sensory deficits, the lesion may involve the cavernous sinus, and targeted imaging of the cavernous sinus region is indicated [10].

### Summary

#### Examination

Assess direct and consensual light responses and compare pupil size in light and darkness.

#### Definition

Unequal pupil size greater than 1 mm.

#### ED Pearl

Painful anisocoria should be considered a PCOM aneurysm until proven otherwise.

**Table 1:** Clinical associations and common causes of anisocoria

Association	Key Clinical Findings	Common Causes / Clinical Context
Physiologic anisocoria	Stable difference < 1 mm; normal light response	Normal variant
Horner syndrome	Miosis + mild ptosis ± anhidrosis	Carotid artery dissection, Pancoast tumor, brainstem stroke
CN III palsy	Dilated pupil + ophthalmoplegia ± ptosis	PCOM aneurysm, tumor, diabetes
Pharmacologic mydriasis	Dilated pupil nonreactive to light	Anticholinergics (atropine, scopolamine), ipratropium exposure
Adie tonic pupil	Dilated pupil with slow constriction to near stimulus	Ciliary ganglion dysfunction (idiopathic)
Opioid intoxication	Bilateral pinpoint pupils with central nervous system depression	Opioid overdose
Pontine lesion	Pinpoint pupils with preserved light response	Pontine hemorrhage or infarction

Table 1: Key clinical findings associated with common etiologies of anisocoria to assist rapid bedside identification of pupillary abnormalities in emergency and neurologic evaluation.

### Adie tonic pupil

#### Description

**Fig. 2:**
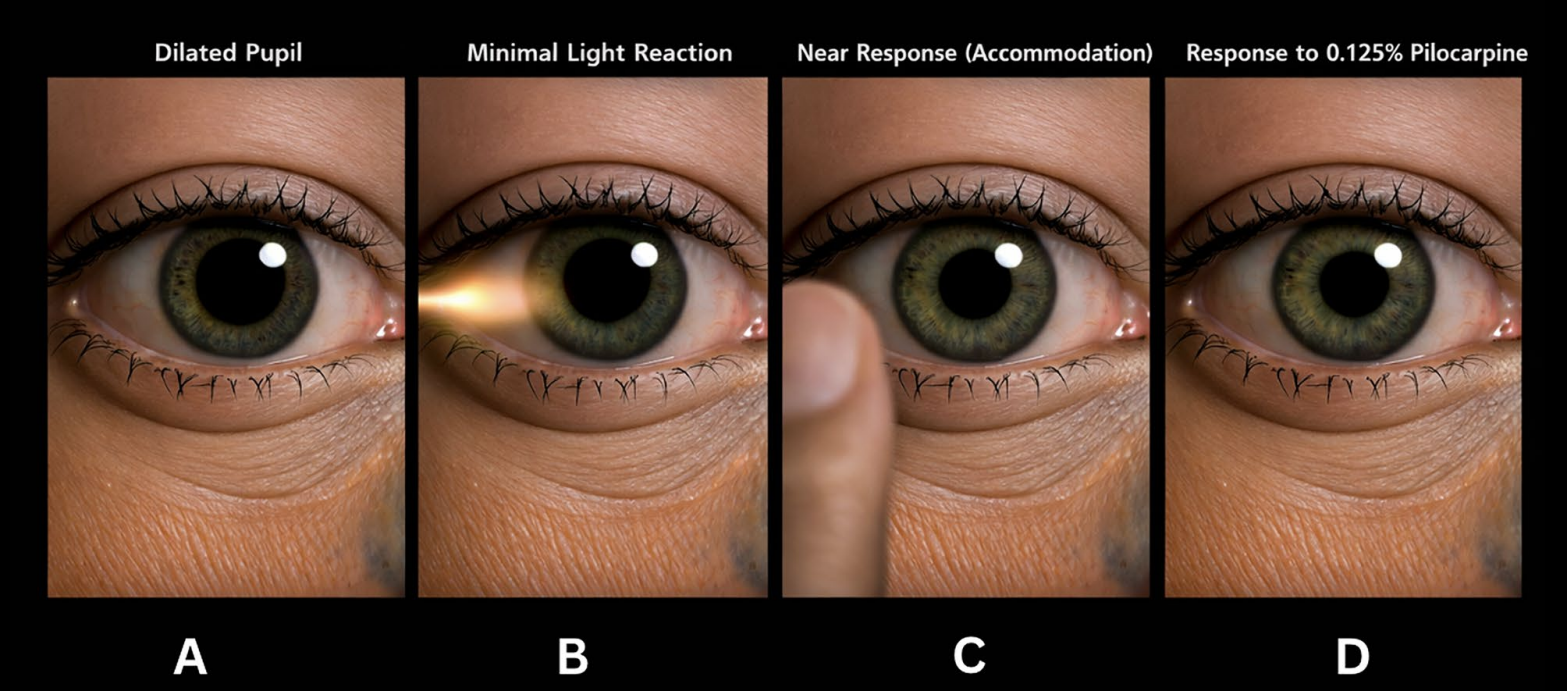
Clinical features of Adie tonic pupil. (**A**) A dilated pupil at rest consistent with parasympathetic denervation. (**B**) Minimal pupillary constriction in response to direct light stimulation. (**C**) Preserved pupillary constriction during near accommodation demonstrating light–near dissociation, a hallmark feature of Adie tonic pupil. (**D**) Constriction following administration of dilute (0.125%) pilocarpine due to denervation supersensitivity

Adie pupil is characterized by a dilated pupil that demonstrates minimal or absent response to light but preserved constriction during accommodation. It is most commonly seen in women in the third or fourth decade of life and is unilateral in approximately 80% of cases [11, 12]. A classic presentation of Adie tonic pupil is illustrated in Fig. 2.

#### Physical examination

On examination, you will test light reactions and the patient’s accommodation reflexes for both pupils. Visual acuity and extraocular movements should also be assessed to exclude CN III palsy.

The affected pupil will appear dilated and has little to no reaction to direct light stimulation. However, the pupil demonstrates slow constriction during near accommodation, a phenomenon referred to as light–near dissociation. The constriction response is typically slow and tonic, and the pupil may redilate gradually after near effort [13].

Low-concentration pilocarpine (0.1–0.125%) may be used to demonstrate cholinergic denervation supersensitivity, which is commonly present in Adie tonic pupils. Following administration, the affected pupil typically constricts more than the unaffected eye because dilute pilocarpine produces little effect in unaffected pupils. However, some pupils affected by CN III palsy may also respond to low-dose pilocarpine, therefore, pharmacologic testing should be interpreted in the context of the overall clinical examination [12].

#### Neuropathophysiology

Adie tonic pupil develops when the ciliary ganglion or its short ciliary nerves are damaged, usually from an inflammatory or post‑infectious process. These structures carry the postganglionic parasympathetic fibers responsible for constricting the pupil and driving accommodation. The preganglionic fibers travel with the CN III and normally synapse in the ciliary ganglion before branching out to the iris and ciliary body. When this ganglion is injured, the output to these targets becomes lost [11, 12].

A key anatomical detail is that the parasympathetic system sends more fibers to the ciliary body than to the iris sphincter. When the ciliary ganglion is injured, the parasympathetic fibers that control the pupil regenerate unpredictably. Because the accommodation pathway has a much larger fiber supply, most of the regrowth follows those routes, and some of those fibers reconnect with the iris sphincter. The result is the characteristic light–near dissociation: a weak or absent light response, but a slow, sustained constriction with near effort [11, 12]. Because the denervated iris sphincter becomes more sensitive to neurotransmitters, the pupil develops denervation hypersensitivity. As a result, even very dilute cholinergic drops can trigger an exaggerated constriction, which is a useful diagnostic feature [12].

#### Clinical relevance and causes

Adie pupil is most often idiopathic, but it may occur following viral infection, inflammation, trauma, or injury to the ciliary ganglion. It may also be associated with Holmes–Adie syndrome, a condition characterized by tonic pupil and diminished deep tendon reflexes. Although typically benign, it is important to distinguish Adie pupil from other causes of a dilated pupil, particularly pharmacologic mydriasis and compressive CN III palsy [13].

#### Summary

##### Examination

Dilated pupil with poor response to light but slow constriction with near effort.

##### Definition

Postganglionic parasympathetic denervation producing a tonic pupil with light–near dissociation.

##### ED Pearl

A unilateral dilated pupil with preserved near constriction and otherwise normal neurologic examination strongly suggests Adie pupil rather than an emergent compressive lesion.

### Argyll robertson pupil

#### Description

Argyll Robertson pupil is classically described as “accommodates but does not react.” Clinically, it presents as small, irregular pupils with a preserved accommodation response. The pupils are typically miotic, irregular in outline, and demonstrate light–near dissociation, reacting poorly to light but constricting normally during accommodation. The abnormality is usually bilateral and symmetric, and visual acuity remains intact [14].

#### Physical examination

**Fig. 3:**
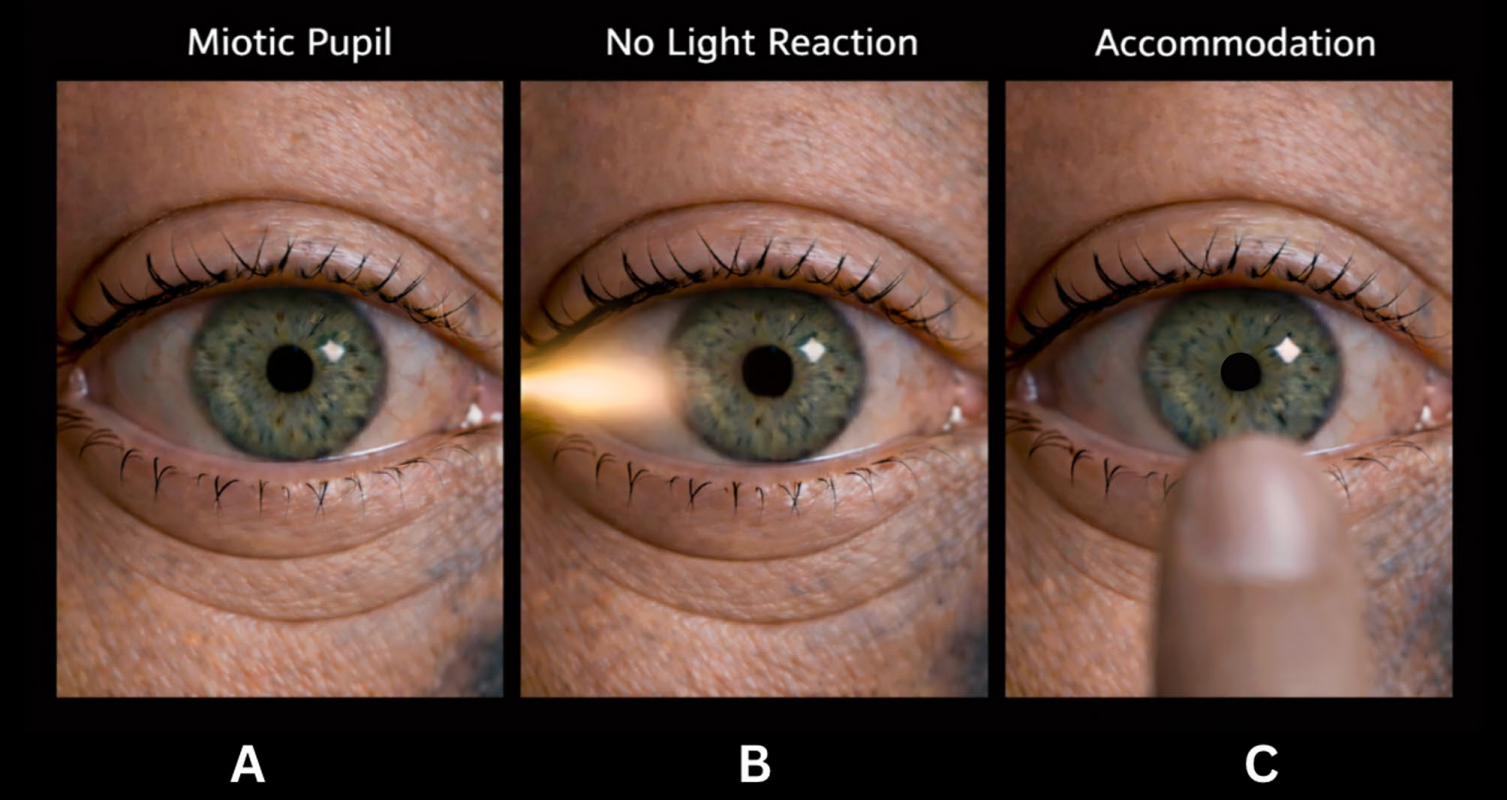
Illustration of Argyll Robertson pupils demonstrating light–near dissociation. (**A**) Baseline examination shows a miotic pupil. (**B**) There is no pupillary constriction in response to direct light stimulation. (**C**) Pupillary constriction occurs during accommodation to a near target, demonstrating preserved near response despite absent light reflex

On examination, the pupils appear small and irregular and show minimal or absent constriction to direct light stimulation. However, they constrict appropriately when the patient focuses on a near object, demonstrating a preserved accommodation response. This pattern of light–near dissociation is the defining clinical feature. A classic presentation of Argyll Robertson pupil is illustrated in Fig. 3.

#### Neuropathophysiology

Light–near dissociation occurs because the neural pathways for the pupillary light reflex and the accommodation reflex diverge within the midbrain. Fibers mediating the light reflex enter the dorsal brainstem and synapse in the pretectal nuclei, whereas fibers mediating the near response ascend to the Edinger–Westphal nucleus from a more ventral pathway. Lesions involving the pretectal region, posterior commissure, or dorsal rostral midbrain disrupt the light reflex pathway while sparing the accommodation pathway, resulting in preserved near constriction despite absent light response [15].

#### Clinical relevance and causes

Argyll Robertson pupil is classically associated with neurosyphilis, particularly in patients with tabes dorsalis or general paresis. Although less common in modern clinical practice due to widespread antibiotic treatment, it remains an important historical and diagnostic finding. Similar pupillary abnormalities may also occur in other lesions of the dorsal midbrain, although neurosyphilis remains the classic association [15].

#### Summary

##### Examination

Small, irregular pupils that constrict with near accommodation but not to light stimulation.

##### Definition

Light–near dissociation caused by lesions of the dorsal midbrain affecting the pupillary light reflex pathway.

##### ED Pearl

Bilateral small pupils with preserved near response should raise suspicion for neurosyphilis or dorsal midbrain pathology.

### RAPD

#### Description

A RAPD occurs when one eye transmits a weaker sensory signal through the afferent limb of the pupillary light reflex. Under normal conditions, light directed into either eye produces bilateral pupillary constriction because afferent input from the retina travels through the optic nerve to midbrain centers that activate both pupils. The presence of RAPD typically indicates unilateral or asymmetric disease affecting the retina or optic nerve [16].

**Fig. 4:**
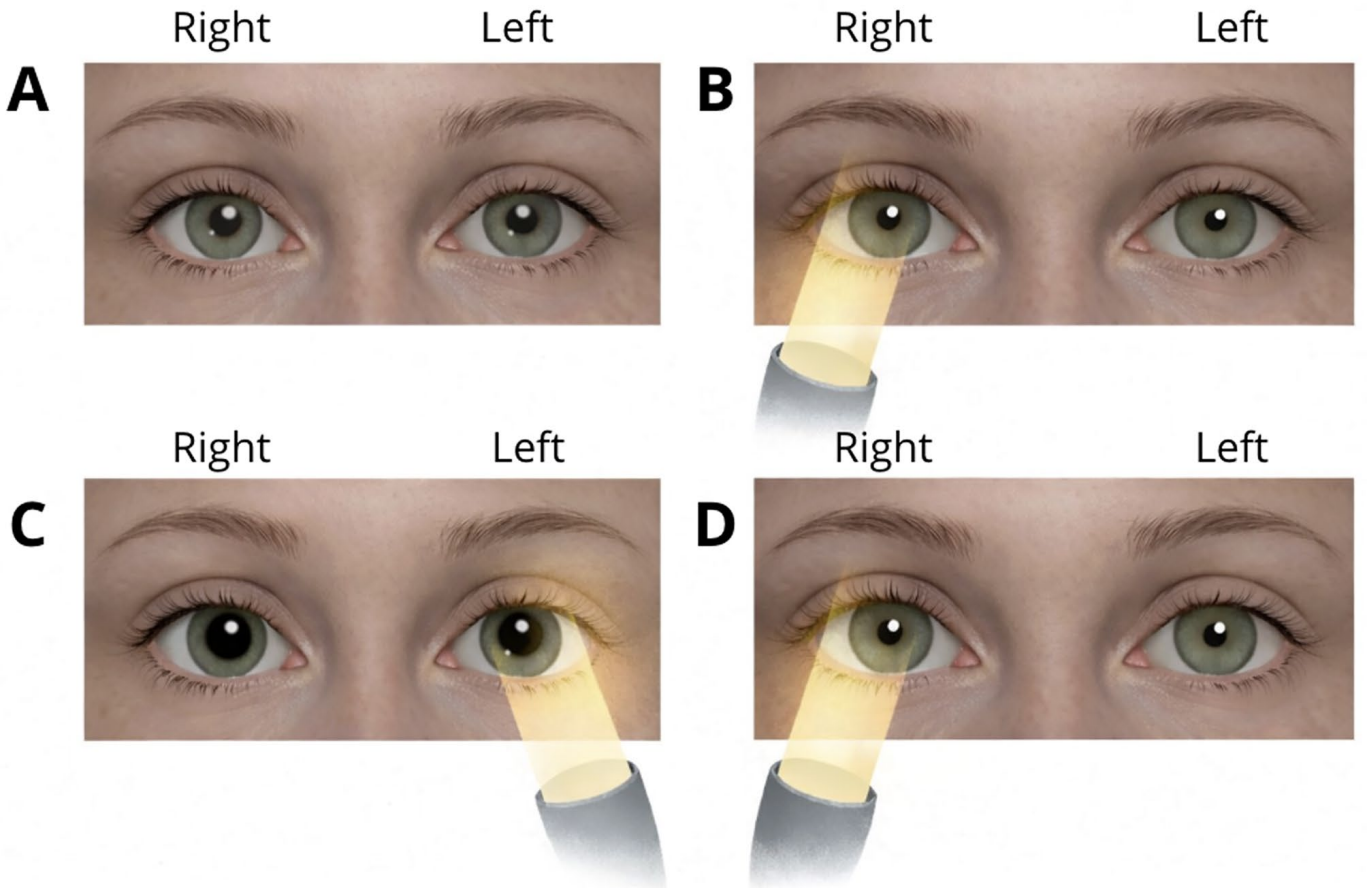
RAPD demonstrated using the swinging flashlight test. (**A**) In ambient light, both pupils are equal in size. (**B**) When light is directed into the unaffected (right) eye, both pupils constrict due to intact direct and consensual responses. (**C**) When the light is swung to the affected (left) eye, reduced afferent input causes both pupils to paradoxically dilate. (**D**) When the light is returned to the normal eye, both pupils constrict again

When this pathway is impaired in one eye, the pupillary response becomes asymmetric. As a result, illumination of the affected eye produces less constriction of both pupils compared with illumination of the normal eye. This phenomenon is classically referred to as the Marcus Gunn pupil [16]. A classic presentation of RAPD is illustrated in Fig. 4.

#### Physical examination

RAPD is detected using the swinging flashlight test. In a dimly lit room, the examiner asks the patient to fixate on a distant object to prevent accommodative constriction. A light is directed into one eye for several seconds and then rapidly moved to the opposite eye while observing the pupillary response. When the normal eye is illuminated, both pupils constrict normally. When the light is moved to the affected eye, both pupils paradoxically dilate rather than constrict [4].

#### Neuropathophysiology

The pupillary light reflex consists of an afferent pathway and an efferent pathway. Light detected by retinal photoreceptors travels through the optic nerve, optic chiasm, and optic tract to the pretectal nuclei of the midbrain. These nuclei then project bilaterally to the Edinger–Westphal nuclei, which send parasympathetic fibers through the CN III to the ciliary ganglion and ultimately the iris sphincter muscles.

Because each eye sends signals to both sides of the midbrain, illumination of either eye normally produces equal constriction of both pupils. When disease affects the retina or optic nerve of one eye, the total afferent signal entering the reflex arc is reduced. Consequently, both pupils constrict less when light is directed toward the affected eye, creating the characteristic relative afferent pupillary defect [4, 16].

#### Clinical relevance and causes

RAPD most commonly reflects optic nerve pathology, although severe retinal disease may also produce this finding.

Common causes include:

- Optic neuritis.
- Ischemic optic neuropathy.
- Retinal detachment.
- Central retinal artery or vein occlusion.
- Optic nerve tumors.
- Advanced glaucoma.

Lesions involving the optic tract or pretectal region may also produce RAPD when the afferent input becomes asymmetric. Importantly, RAPD does not itself cause anisocoria; the pupils are typically equal in size at rest. Instead, the abnormality becomes apparent only during dynamic testing of the light reflex [4, 16].

#### Summary

##### Examination

Swinging flashlight test shows paradoxical dilation when light is directed toward the affected eye.

##### Definition

An asymmetric defect in the afferent limb of the pupillary light reflex, usually involving the retina or optic nerve.

##### ED Pearl

RAPD strongly suggests optic nerve or severe retinal pathology and should prompt evaluation for conditions such as optic neuritis, retinal detachment, or retinal vascular occlusion.

### Horner syndrome

#### Description

Horner syndrome is characterized by anisocoria in which the affected pupil is smaller than the contralateral pupil while retaining normal light reactivity. The condition results from disruption of the sympathetic pathway supplying the eye.

**Fig. 5:**
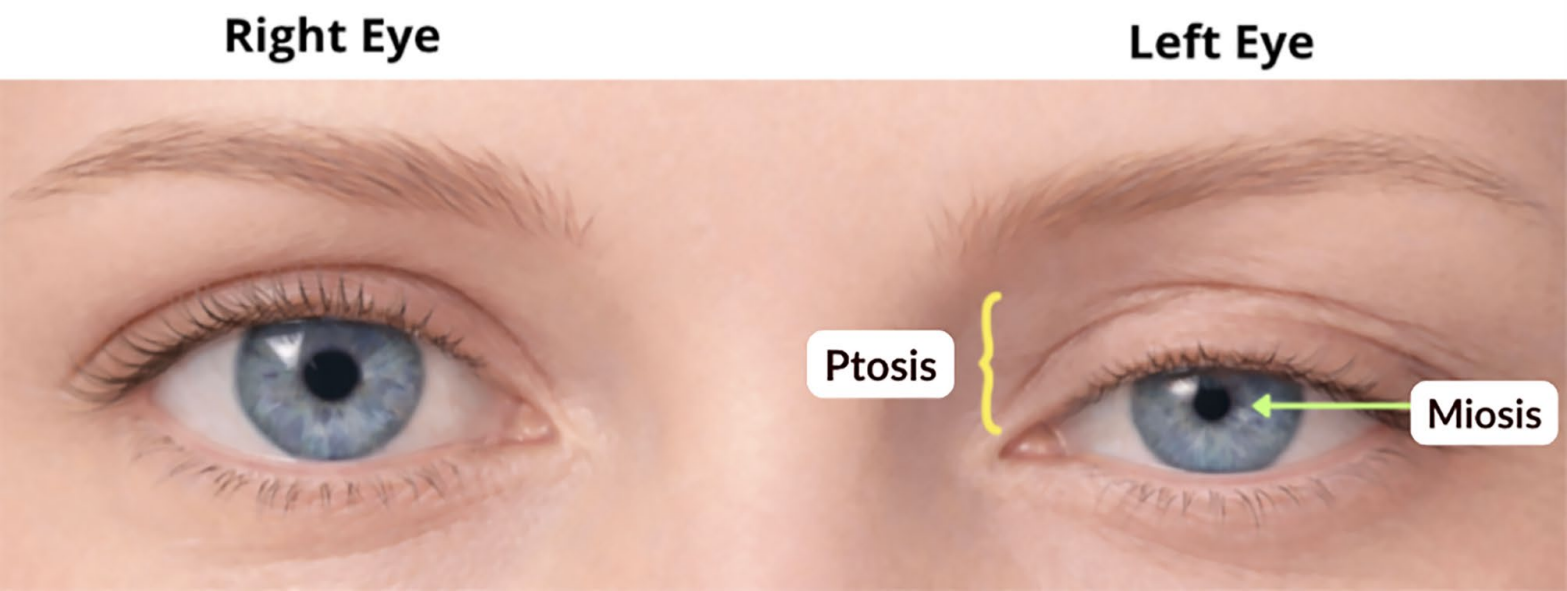
Horner syndrome. Clinical illustration demonstrating mild ptosis and miosis of the left eye due to disruption of the sympathetic pathway. The contralateral right eye maintains normal pupil size and eyelid position for comparison

Patients typically demonstrate mild ptosis of the upper eyelid, ipsilateral facial anhidrosis and impaired temperature regulation may also occur, depending on the location of the lesion [17]. The ptosis seen in Horner syndrome is subtle, in contrast to the more pronounced ptosis observed in CN III palsy, where the levator palpebrae muscle is affected. A classic presentation of Horner syndrome is illustrated in Fig. 5.

#### Physical examination

On examination, the affected pupil appears constricted (miosis) and demonstrates preserved reactivity to light. The degree of anisocoria is often more pronounced in dim lighting, because the abnormal pupil cannot dilate appropriately.

Additional findings may include mild upper eyelid ptosis, slight elevation of the lower eyelid, narrowing of the palpebral fissure, and ipsilateral anhidrosis.

#### Neuropathophysiology

Horner syndrome results from interruption of the oculosympathetic pathway, which normally provides sympathetic innervation to the iris dilator muscle and eyelid smooth muscle. Disruption of this pathway results in loss of sympathetic tone, leading to unopposed parasympathetic constriction of the pupil [17, 18].

This pathway consists of a three-neuron chain:

- First-order neuron: hypothalamus → brainstem → ciliospinal center (C8–T2).
- Second-order neuron: spinal cord → superior cervical ganglion.
- Third-order neuron: superior cervical ganglion → along the internal carotid artery → cavernous sinus → iris dilator muscle.

#### Clinical relevance and causes

Horner syndrome may result from lesions affecting any portion of the sympathetic pathway. The presence of facial anhidrosis may help localize the lesion. Proximal lesions involving the first-order neuron are more likely to produce anhidrosis, whereas distal lesions along the internal carotid artery often spare facial sweating. Anhidrosis is more commonly associated with proximal (first-order) lesions, whereas lesions along the internal carotid artery (third-order neuron) often spare facial sweating [17].

Important causes include:

- Internal carotid artery dissection.
- Pancoast tumor.
- Brainstem stroke, such as Wallenberg syndrome.
- Cavernous sinus pathology.

#### Summary

##### Examination

Unilateral miosis with preserved light reactivity, mild ptosis, and possible facial anhidrosis.

##### Definition

Disruption of the sympathetic pathway to the eye, producing loss of pupillary dilation and mild eyelid ptosis.

##### ED Pearl

Acute neck pain with Horner syndrome should raise concern for carotid artery dissection until proven otherwise.

### CN III palsy

#### Description

CN III palsy typically presents with ptosis, diplopia, and abnormal eye positioning, often described as a “down and out” eye. This occurs because the lateral rectus (CN VI) and superior oblique (CN IV) muscles act unopposed when the muscles normally innervated by CN III become dysfunctional [18].

**Fig. 6:**
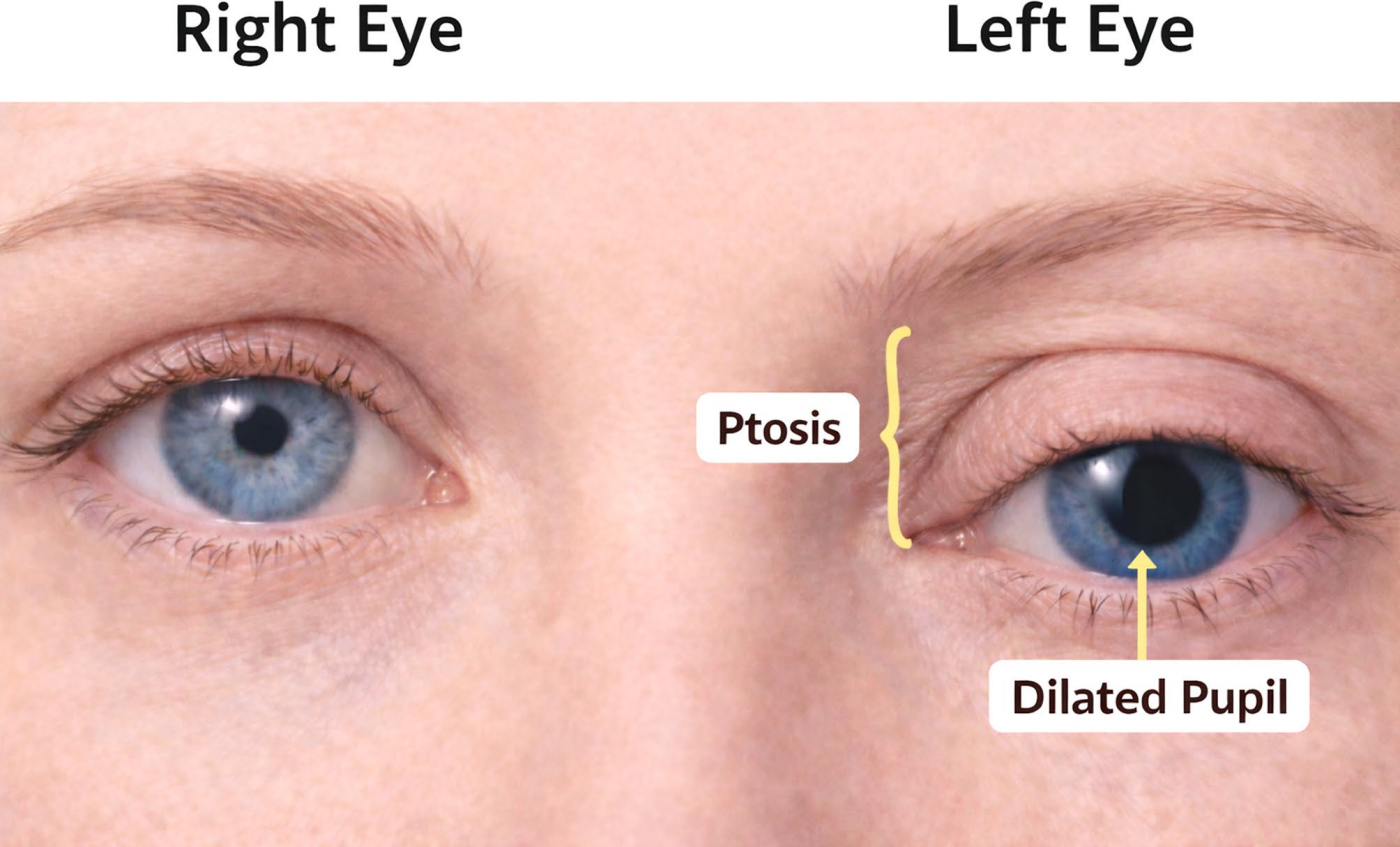
Cranial Nerve III Palsy. Clinical illustration demonstrating marked ptosis and ipsilateral mydriasis. The left eye is affected, showing pupillary dilation due to parasympathetic fiber involvement, while the right eye demonstrates normal pupillary size and eyelid position

CN III controls the superior rectus, medial rectus, inferior rectus, and inferior oblique muscles and provides motor input to the levator palpebrae superioris, which elevates the upper eyelid. It also carries parasympathetic fibers responsible for pupillary constriction [18]. A classic example of CN III palsy is illustrated in Fig. 6.

### Physical examination

Evaluation begins with a complete cranial nerve examination, with particular attention to extraocular movements and pupillary response.

Extraocular movements are assessed by having the patient follow the examiner’s finger through the H-pattern, which tests coordinated movement of the extraocular muscles. In CN III palsy, the affected eye demonstrates limited adduction, elevation, and depression, and typically rests in a downward and lateral position.

Patients commonly exhibit marked ptosis due to dysfunction of the levator palpebrae muscle. Pupillary examination should assess size, symmetry, and reactivity to light. A dilated, poorly reactive pupil suggests involvement of the parasympathetic fibers traveling with CN III [10].

### Neuropathophysiology

CN III contains both motor fibers controlling extraocular muscles and parasympathetic fibers responsible for pupillary constriction.

The parasympathetic fibers run superficially along the nerve, making them particularly vulnerable to external compression. In contrast, microvascular ischemia typically affects the central motor fibers while sparing the superficial parasympathetic fibers. This difference explains why some cases present with pupil involvement, while others demonstrate pupil sparing [18].

### Clinical relevance and causes

CN III palsy may result from compressive lesions or microvascular ischemia.

Important compressive causes include:

- PCOM aneurysm.
- Intracranial tumors.
- Cavernous sinus pathology.

Microvascular ischemic causes are most often associated with vascular risk factors that include:

- Diabetes mellitus.
- Hypertension.
- Atherosclerosis.

Historically, the “rule of the pupil” suggested that pupil-involving palsies were compressive and pupil-sparing palsies were ischemic. However, because vascular imaging is widely available, many clinicians now obtain CTA or MRA to exclude aneurysm or other structural causes in suspected cases [10].

#### Summary

##### Examination

Ptosis, “down and out” eye deviation, impaired extraocular movements on H-pattern testing, and possible pupillary dilation.

##### Definition

Dysfunction of CN III impairing extraocular movement, eyelid elevation, and pupillary constriction.

##### ED Pearl

A dilated pupil in the setting of CN III palsy should raise concern for a compressive lesion, particularly a posterior communicating artery aneurysm, and warrants urgent vascular imaging.

### Hutchinson pupil

#### Description

**Fig. 7:**
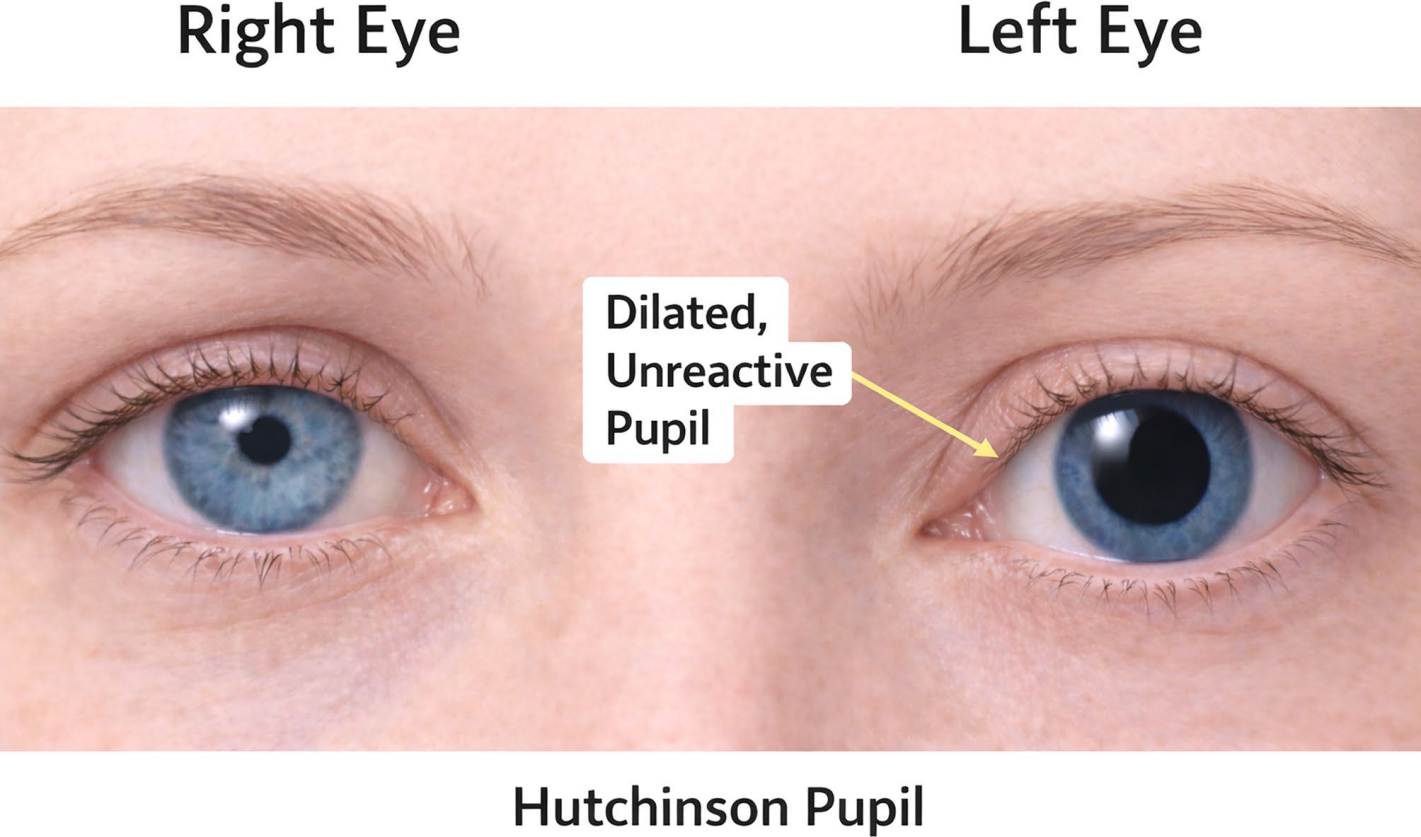
Hutchinson pupil. Clinical illustration demonstrating a unilateral dilated, non-reactive pupil caused by compression of CN III, often due to intracranial mass effect and impending uncal herniation. The left eye is affected, showing marked mydriasis, while the right eye demonstrates normal pupillary size and reactivity for comparison

Hutchinson pupil refers to a unilateral dilated and non-reactive pupil caused by compression of the CN III, most commonly in the setting of uncal herniation from intracranial mass effect. As intracranial pressure increases, the medial temporal lobe compresses the ipsilateral CN III, producing a fixed dilated pupil [19, 20]. A classic example of this finding is shown in Fig. 7.

#### Physical examination

On examination, the affected pupil is dilated and poorly reactive or non-reactive to light. Patients may also demonstrate ptosis or impaired extraocular movements due to involvement of CN III. In severe cases, Hutchinson pupil is associated with progressive neurologic deterioration, including decreased level of consciousness.

#### Neuropathophysiology

The parasympathetic fibers responsible for pupillary constriction travel along the peripheral portion of CN III, making them particularly susceptible to external compression. When these fibers are disrupted, the iris sphincter loses its ability to constrict, resulting in pupillary dilation [19].

#### Clinical relevance and causes

Hutchinson pupil most commonly occurs in conditions producing intracranial mass effect, including:

- Epidural hematoma.
- Subdural hematoma.
- Temporal lobe mass lesions.
- Severe traumatic brain injury.

In rare cases, a false localizing sign may occur due to the Kernohan–Woltman notch phenomenon. During significant intracranial mass effect and uncal herniation, the contralateral cerebral peduncle may be compressed against the tentorial edge, injuring corticospinal tract fibers. This can produce ipsilateral hemiparesis, meaning motor weakness appears on the same side as the intracranial lesion, rather than the contralateral side typically expected. This paradoxical deficit is therefore considered a false localizing sign, as the neurological findings do not correspond to the true location of the lesion [19, 20].

#### Summary

##### Examination

Unilateral fixed dilated pupil with poor or absent light response.

##### Definition

Compression of CN III from intracranial mass effect, classically due to uncal herniation.

##### ED Pearl

A new unilateral fixed dilated pupil in a patient with head trauma or declining mental status should raise concern for uncal herniation and requires emergent neuroimaging.

**Table 2:** Clinically Significant Pupillary Findings in Emergency and Urgent Care Settings

Pupillary Finding	Key Bedside Features	High-Risk Etiologies
Physiologic anisocoria	Stable difference < 1 mm, normal light response	Benign physiologic variation
Adie tonic pupil	Dilated pupil with poor light response but preserved near constriction	Ciliary ganglion dysfunction
Argyll Robertson pupil	Bilateral miotic pupils with preserved accommodation but absent light response	Neurosyphilis, dorsal midbrain lesion
RAPD	Paradoxical dilation on swinging flashlight test	Optic neuritis, retinal detachment
Horner syndrome	Miosis, mild ptosis, ± anhidrosis	Carotid artery dissection, brainstem stroke
CN III palsy	Ptosis, ophthalmoplegia, ± dilated pupil	PCOM aneurysm
Hutchinson pupil	Unilateral fixed dilated pupil	Uncal herniation from intracranial mass

Table 2: Summary of key pupillary abnormalities encountered in emergency and urgent care settings, highlighting characteristic bedside findings and associated neurologic or systemic etiologies.

### Emergency department clinical takeaways

Pupillary abnormalities are uncommon but clinically important findings in emergency and urgent care settings. Their diagnostic significance increases when they occur in the context of headache, altered mental status, trauma, or focal neurologic deficits.

#### Three bedside principles are particularly important

1. Determine which pupil is abnormal.

- Comparison of pupil size in bright and dim illumination helps determine whether the abnormality reflects parasympathetic dysfunction (dilated pupil) or sympathetic dysfunction (constricted pupil).

2. Associated neurologic findings guide urgency.

- Ptosis, ophthalmoplegia, or altered mental status should raise concern for serious neurologic pathology such as compressive aneurysm, carotid artery dissection, or intracranial mass effect.

3. Serial examinations are critical.

- Changes in pupil size or reactivity over time may signal evolving neurologic injury and should prompt urgent reassessment and imaging.

### Example documentation of pupillary examination

Pupillary examination revealed symmetric pupils measuring 3 mm bilaterally with brisk reactivity to light. No anisocoria, ptosis, or abnormal extraocular movements were observed. Cranial nerve examination was otherwise normal. Findings did not suggest acute CN III palsy or intracranial mass effect.

Patients were instructed to return immediately for worsening headache, confusion, visual changes, unequal pupils, or new neurologic symptoms.

## Data Availability

All data discussed in this review are derived from previously published sources cited in the reference list. Figures included in this manuscript were generated using artificial intelligence–based image generation tools for illustrative and educational purposes and do not depict real patients or identifiable individuals.

## Abbreviations

POCUS: Point-of-care ultrasound
MRI: Magnetic resonance imaging
CT: Computed tomography
MRA: Magnetic resonance angiography
CN: Cranial nerve
CN III: Cranial nerve III
RAPD: Relative afferent pupillary defect
PCOM: Posterior communicating artery
Mm: Millimeter
CTA: Computed tomography angiography
